# Combined analysis of host IFN-γ, IL-2 and IP-10 as potential LTBI biomarkers in ESAT-6/CFP-10 stimulated blood

**DOI:** 10.3389/fmmed.2024.1345510

**Published:** 2024-01-26

**Authors:** Antony M. Rapulana, Thabo Mpotje, Omolara O. Baiyegunhi, Hlumani Ndlovu, Theresa K. Smit, Timothy D. McHugh, Mohlopheni J. Marakalala

**Affiliations:** ^1^ School of Laboratory Medicine and Medical Science, University of Kwazulu-Natal, Durban, Kwazulu-Natal, South Africa; ^2^ Africa Health Research Institute, Durban, Kwazulu-Natal, South Africa; ^3^ Division of Infection and Immunity, UCL Centre for Clinical for Clinical Microbiology, University College London, London, United Kingdom; ^4^ Division of Chemical and System Biology, Department of Integrative Biomedical Sciences, Faculty of Health Sciences, University of Cape Town, Cape Town, South Africa

**Keywords:** tuberculosis, latent tuberculosis infection, diagnostic biomarkers, LTBI (latent TB infection), IP-10 (IFN-γ inducible protein 10), interferon gamma (IFN-γ), IL-2 (interleukin-2), biomarkers

## Abstract

**Background:** Accurate diagnosis of latent tuberculosis infected (LTBI) individuals is important in identifying individuals at risk of developing active tuberculosis. Current diagnosis of LTBI routinely relies on the detection and measurement of immune responses using the Tuberculin Skin Test (TST) and interferon gamma release assays (IGRAs). However, IGRA, which detects *Mycobacterium tuberculosis* specific IFN-γ, is associated with frequent indeterminate results, particularly in immunosuppressed patients. There is a need to identify more sensitive LTBI point of care diagnostic biomarkers. The aim of this study was to assess the validity of early secreted antigen target 6 kDa (ESAT-6) and culture filtrate protein 10 (CFP-10) stimulated plasma to identify additional cytokines and chemokines as potential biomarkers of LTBI.

**Method:** The levels of 27 cytokines and chemokines were measured by Bio-Plex Pro cytokine, chemokine and growth factor assay in ESAT-6 and CFP-10 co-stimulated plasma from 20 LTBI participants with positive IGRA (Quantiferon TB Gold plus) and 20 healthy controls with negative IGRA. Traditional ELISA was used to validate the abundance of the best performing markers in 70 LTBI and 72 healthy participants. All participants were HIV negative.

**Results:** We found that Interleukin 1 receptor antagonist (IL1ra) (*p* = 0.0056), Interleukin 2 (IL-2) (*p* < 0.0001), Interleukin 13 (IL-13) (*p* < 0.0001), Interferon gamma-induced protein 10 (IP-10) (*p* < 0.0001), and Macrophage inflammatory protein-1 beta (MIP1b) (*p* = 0.0010) were significantly higher in stimulated plasma of LTBI compared to healthy individuals. Stimulated plasma IL-2 (cutoff 100 pg/mL), IP-10 (cutoff 300 pg/mL) and IL-13 (5 pg/mL) showed potential in diagnosing LTBI with PPV = 100%, 0.89.4%, and 80.9% and NPV = 86.9%, 0.85.7%, and 84.2%, respectively.

**Conclusion:** Our data shows that co-stimulating whole blood with ESAT-6 and CFP-10 may help distinguish LTBI from healthy individuals. We also identified IL-2 and IP-10 as potential biomarkers that could be added to the currently used IFN-γ release assays in detection of LTBI.

## Introduction

One-third of the world population is estimated to be infected with *Mycobacterium tuberculosis* (*M.tb*), and 5–10 percent of the latently infected individuals will develop active tuberculosis (TB) in their lifetime (WHO and Global, 2020). The WHO has recommended diagnosis and treatment of LTBI to prevent progression to active TB and has set a goal of ≥90% treatment of all TB cases by 2025 (WHO, 2018).

Several studies have shown that measurement of soluble immune mediators such as interferon gamma (IFN-γ) and interleukin-6 (IL-6) in peripheral blood may be used to distinguish the individuals that have been exposed to *M.tb* (Suzukawa et al., 2016; Kumar et al., 2021). These immune mediators play a role in granuloma formation, including recruitment and activation of multiple networks of immune cells that constitute the granuloma (Ferluga et al., 2020). Other studies have explored transcriptional and proteomic biosignatures as potential diagnostic biomarkers of TB or risk of disease progression (Kaforou et al., 2013; Scriba et al., 2017; Walzl et al., 2018).

Current diagnosis of LTBI is based on the tuberculin skin test (TST) or interferon gamma release assays (IGRAs), although they measure different components of the immunological response and are not interchangeable. Both TST and the IGRA have disadvantages which include reduced sensitivity in individuals with immunosuppression, inability to distinguish *M.tb* from other mycobacteria (e.g., *M. Kansasii*) and the failure to distinguish LTBI from active TB (Shim, 2014; Auguste et al., 2017; Goletti et al., 2022). Therefore, we sought to assess the validity of using early secretary antigen target 6 (ESAT-6) and culture filtrate protein 10 (CFP-10) stimulated plasma to distinguish individuals diagnosed with LTBI from healthy (non-LTBI and non-TB) individuals. We report that measurement of the levels of IL-1ra, IL-2, IL-13, and chemokines IP-10 and MCP-1 in ESAT-6 and CFP-10 stimulated plasma may be used to distinguish between healthy and LTBI individuals.

## Materials and methods

### Study design and setting

The study participants were recruited from healthcare facilities (KwaDabeka Clinic and Prince Cyril Zulu Communicable Disease Centre), selected as two of the major out-patient TB treatment facilities in eTheKwini District Kwazulu-Natal (KZN), South Africa. The protocol for this study was reviewed and approved by the University of Kwazulu-Natal Biomedical Research Ethics Committee (BE022/13). All participants provided written informed consent to participate in the study. We recruited 70 LTBI (QuantiFERON (QFT) positive) and 72 healthy (QFT negative) participants with GeneXpert RIF/MTB negative results. All participants were HIV negative. The samples were collected from February 2020 to June 2022. The clinical and demographic characteristics of study participants are shown in Table 1.

**TABLE 1:** Clinical and demographic characteristics of participants.

	Latent TB Infected (LTBI)	Healthy
n	70	72
Age (years)	23 (21–35)	26 (22–34)
Sex		
Male	31 (44%)	42 (58%)
Female	39 (56%)	30 (42%)
HIV status		
Negative	70 (100%)	72 (100%)
Positive	0	0
Ethnicity		
Black	70 (100%)	72 (100%)
Assay		
QFT ELISA	70	72
Multiplex ELISA	20	20
IL-2, IFN-y and IP-10	70	72

### Sample collection

All samples were similarly treated and stimulated immediately after arrival in the laboratory. One milliliter of whole blood was directly collected into each of 4 QFT-gold plus tubes as per manufacturer’s instruction (QIAGEN, Germantown, MD, United States) and incubated for 20 h in 37°C, following incubation, plasma was collected by centrifuging the whole blood at 1,000×g for 10 min. The plasma derived from each of the QFT tubes was aliquoted and stored at −80°C until use. GeneXpert diagnostic testing was conducted on sputum samples collected from the participants as Standard of Care (SOC) at the National Health Laboratory Services, Durban, South Africa, according to the manufacturer’s instructions (Cepheid, Sunnyvale, CA, United States).

### Immunoassays

Interferon gamma (IFN-ƴ) levels in plasma were determined using the Quantiferon TB Gold ELISA (QIAGEN, Germantown, MD, United States) kit according to the manufacturer’s instruction and the results were classified as positive defined by IFN-γ produced in either or both TB1 and/or TB2 minus the NIL tube (without any *M.tb* specific stimulants), negative, or indeterminate using the manufacturer’s software (QIAGEN, Germantown, MD, United States).

### Measurement of plasma cytokines by multiplex ELISA

The analyses of 27 host biomarkers (FGF basic, Eotaxin, G-CSF, GM-CSF, IFN-γ, IL-1β, IL-1ra, IL-2, IL-4, IL-5, IL-6, IL-7, IL-8, IL-9, IL-10, IL-12 (p70), IL-13, IL-15, IL-17A, IP-10, MCP-1 (MCAF), MIP-1a, MIP-1β, PDGF-BB, RANTES, TNF-α and VEGF) in TB antigen-stimulated (TB1) plasma of LTBI and healthy individuals was done using the Bio-Plex Pro Human Cytokine 27-plex Assay (Bio-Rad Laboratories, Hercules, CA, United States). Prior to the assay, all stimulated plasma were diluted 1:1 with the kit serum matrix to ensure accurate measurement of host biomarkers. Assays were performed as per the manufacturer’s instructions and results obtained with the Bio-Plex 200 plate reader and Bio-Plex-Manager Software version 6. The sensitivity of the kit was 0.2–25.6 pg/mL for each of the 27 cytokines measured. A 5-parameter logistic regression (5 PL) formula was used to generate the standard curves for each cytokine to interpolate the concentration of cytokines in the samples. Cytokines for which expression did not achieve the limit of detection were reported as zero.

### IFN-γ, IL-2 and IP-10 ELISA

Frozen plasma aliquots of each participant’s unstimulated (nil) and TB antigen-stimulated plasma (TB1) were thawed and the levels of IL-2, IP-10 and IFN-γ were evaluated using the Human IL-2, Human IP-10 and Human IFN-γ ELISA sets (BD OptEIA, BD Biosciences, San Diego, CA, United States), respectively and performed according to the manufacturer’s instructions. Prior to the assay, plasma for IL-2 analysis were diluted 1:2, and plasma for IFN-γ and IP-10 analysis were diluted 1:4 with sample diluent to ensure accurate measurement of cytokines levels.

### Receiver operator characteristics analyses

We performed receiver operator characteristics (ROC) curve analysis of IL-1ra, IL-2, IP-10, INF-γ, IL-13, and MIP-1b based on the multiplex ELISA data comparing stimulated Healthy and LTBI participants. For all the ROC curves, sensitivity, specificity, and positive and negative predicitive values were determined. We also performed the ROC analysis of IL-2, IP-10, and INF-γ based on traditional ELISA data. We performed combined IL-2, IP-10, and INF-γ, combined IL-2 and IP-10 ROC curves using pROC package to define marker combinations of the tested stimulated plasma cytokines and chemokines.

### Statistical analysis

Statistical analyses were done in GraphPad Prism 9 software (CA, United States) and ROC and summary ROC curves were done using R software version 4.2.1. To identify potential cytokines, chemokines and human growth factor as biomarker that can be used to distinguish healthy and LTBI individuals, the data that were not normally distributed were log transformed and normalized, and a principal component analysis (PCA) performed. Mann-Whitney non-parametric test was used to determine significant differences between 2 unpaired groups. One-way ANOVA was done to compare multiple sample groups, followed by a multiple comparison post-test using Tukey’s test.

## Results

### ESAT-6 and CFP-10 stimulated plasma cytokines distinguish between healthy and LTBI individuals

**FIGURE 1:**
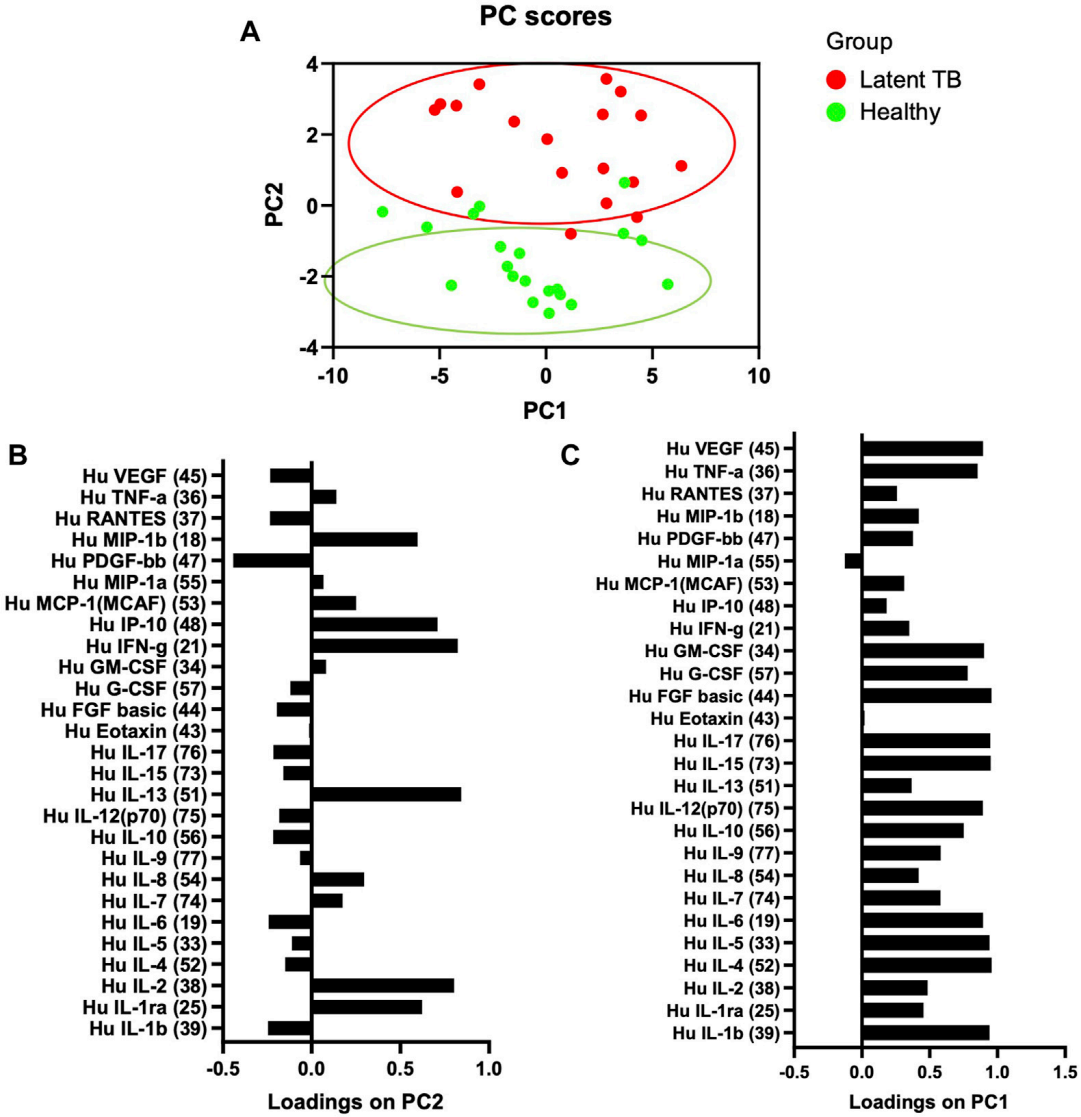
Principal component analysis (PCA) of the association of ESAT-6 and CFP-10 stimulated plasma cytokines between healthy and latent tuberculosis infection groups. **(A)** Stimulated plasma cytokines separate according to whether individuals are healthy or latent TB infected. Each dot represents a participants score on the loading components. **(B,C)** PC2 was used to define the association between stimulated plasma cytokines and whether individuals were healthy or LTBI.

Twenty-seven cytokines and chemokines were measured in ESAT-6 and CFP-10 stimulated plasma from 20 LTBI and 20 healthy participants to screen for biomarkers with a potential to differentiate healthy from LTBI individuals. To determine if there was an association of the cytokines and LTBI, we performed a multivariate principal component analysis (PCA) using unsupervised clustering. Proteins concentrations that segregated according to LTBI, along PC2 (Figure 1A), were associated with higher abundance of cytokines such as IL-1ra, IL-2, IL-13, IFN-y, IP-10, and MIP-1b (Figure 1B), while all other cytokines were loaded on PC1 (Figure 1C). These data suggest that the cytokines might serve as potential biomarkers in distinguishing LTBI from healthy individuals.

### Stimulation of LTBI plasma results in increased cytokine profile when compared to stimulated healthy plasma

**FIGURE 2:**
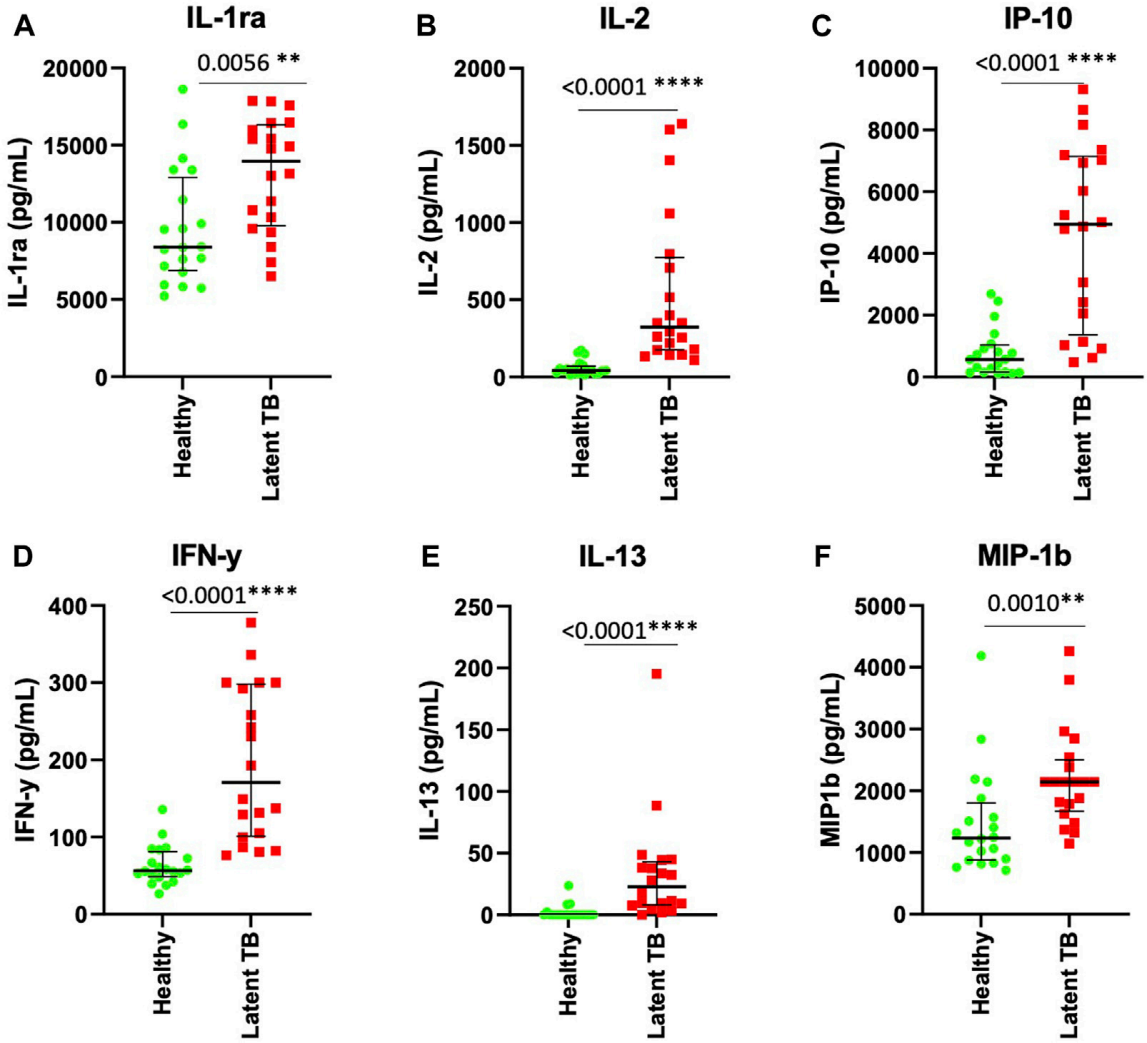
IL-1ra, IL-2, IP-10, IFN-y, IL-13 and MIP-1b are elevated in stimulated plasma from latent tuberculosis infected participants. Each dot represents a participant sample. **(A)** IL-1ra, **(B)** IL-2, **(C)** IP-10, **(D)** IFN-y, **(E)** IL-13, and **(F)** MIP-1b were measured in pg/mL. The significant differences were identified as those values that were less than *p* < 0.05. The data was analyzed using Mann-Whitney non-parametric *t*-test. The error bars show the median and IQR.

Following the identification of biomarkers that were associated with LTBI in stimulated ESAT-6 and CFP-10 plasma, we investigated whether there was any significant difference in biomarkers between LTBI and healthy individuals. IL-1ra (*p* = 0.0056), IL-2 (*p* < 0.0001), IP-10 (*p* < 0.0001), IFN-y (*p* < 0.0001), IL-13 (*p* < 0.0001), and MIP-1b (*p* = 0.0010) were significantly higher (*p* < 0.05) in ESAT-6 and CFP-10 stimulated plasma of LTBI compared to healthy participants, (Figures 2A–F). Abundance of all other cytokines and chemokines measured in stimulated plasma are listed in Supplementary Table S1.

### ROC analysis shows that the inflammatory cytokines (IL-2, IFN-γ, IP-10) have good discriminatory power between the stimulated LTBI and healthy plasma

**FIGURE 3:**
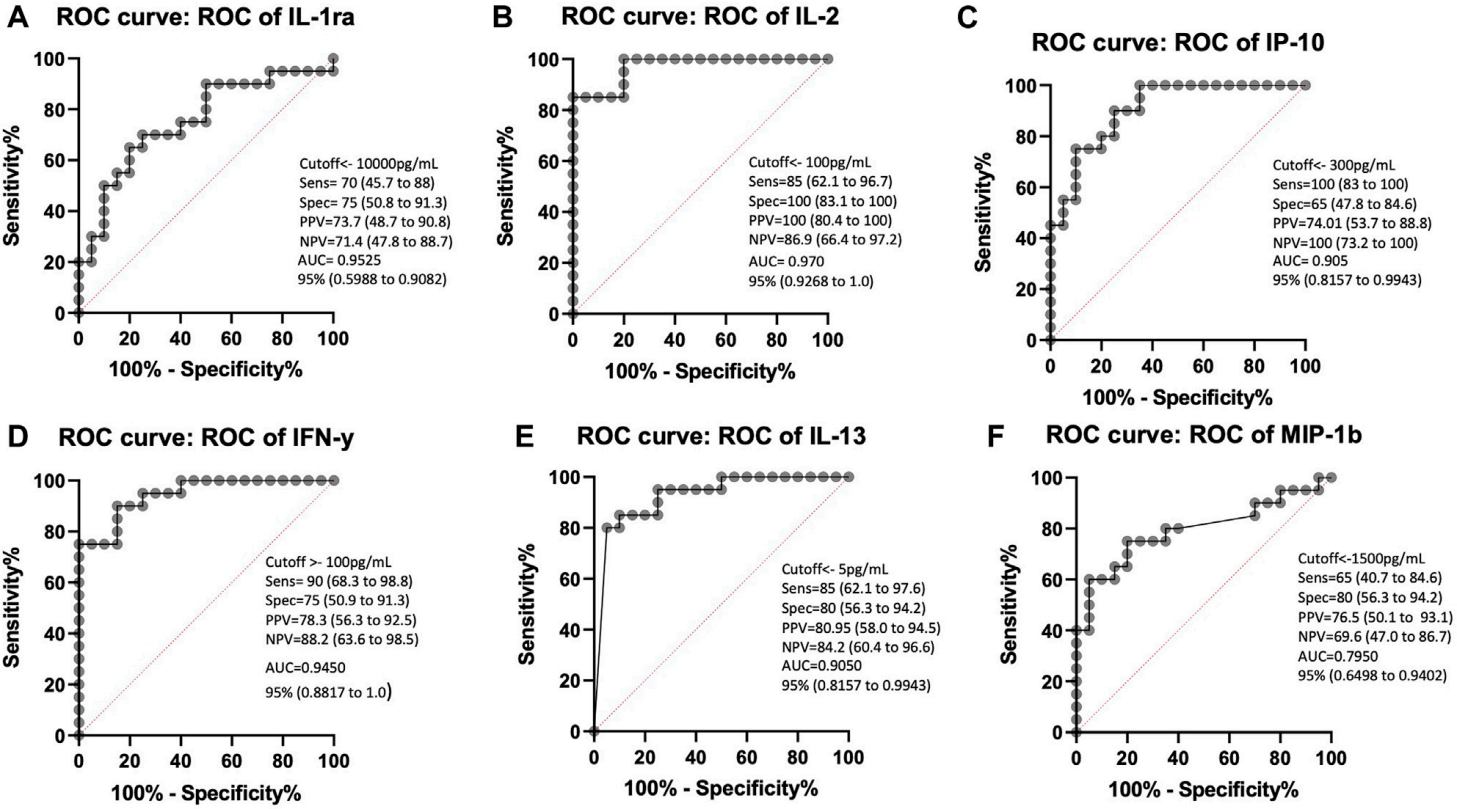
ROC curve characteristics for IL-1ra, IL-2, IP-10, IFN-γ, IL-13 and MIP-1b as discriminatory markers of LTBI. AUC, sensitivity, specificity, PPV, and NPV indicate the power of **(A)** IL-1ra, **(B)** IL-2, **(C)** IP-10, **(D)** IFN-γ, **(E)** IL-13, and **(F)** MIP-1b which are reported and indicate the power of these stimulated plasma biomarkers for discriminating between those who are healthy and those with LTBI.

We performed ROC analysis to assess the power of IL-1ra, IL-2, IP-10, IL-13, IFN-y and MIP-1b as biomarkers in ESAT-6 and CFP10 stimulated plasma to distinguish between LTBI and healthy individuals IL-1ra, IL-2, IP-10, IL-13, IFN-y and MIP-1b showed good area under curve (AUC) results, (AUC = 0.9525, AUC = 0.9700, AUC = 0.9050, AUC = 0.9450, AUC = 0.9050, and AUC = 0.7950). Stimulated plasma IL-1ra at the cutoff of 10 ng/mL had a positive predictive value (PPV) of 73.7% and a negative predictive value (NPV) of 71.4% (Figure 3A). Stimulated plasma IL-2 (cutoff 100 pg/mL), IP-10 (cutoff 300 ng/mL) and IL-13 (5 pg/mL) showed potential in diagnosing LTBI with PPV = 100%, 89.4%, and 80.9% and NPV = 86.9%, 85.7%, and 84.2, respectively (Figures 3B, C, E). IFN-γ had a sensitivity of 90% and specificity of 75% with 100 pg/mL cut-off value (PPV = 78%, NPV = 88%) (Figure 3D). Stimulated MIP-1b had PPV and NPV of 76.5% and 69.6%, respectively (Figure 3F).

### *Mtb*-specific antigens induce significant IP-10, IL-2 and IFN- γ production in LTBI plasma

**FIGURE 4:**
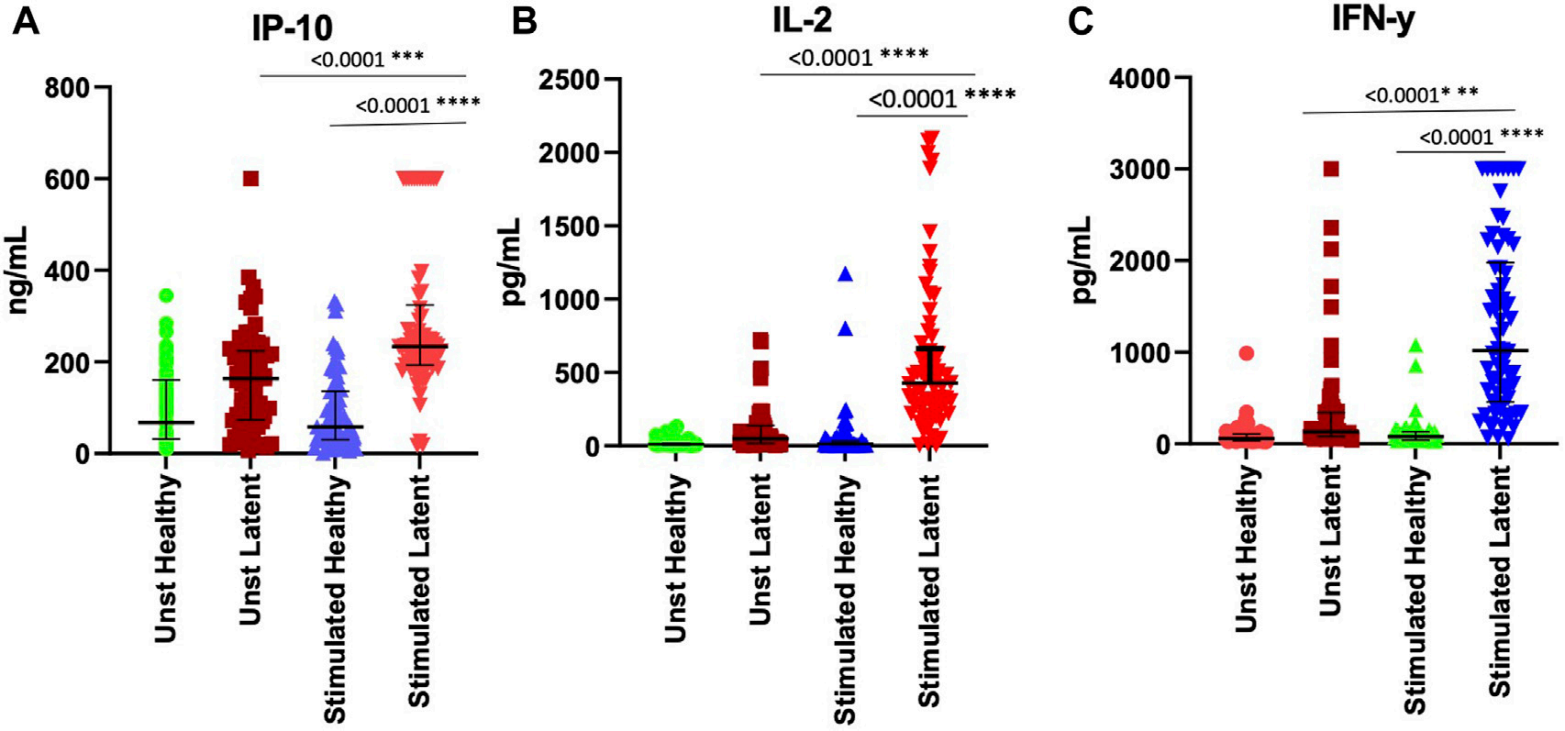
Stimulation of whole blood with ESAT-6 and CFP-10 differentiate healthy and LTBI participants. **(A)** IP-10, **(B)** IL-2, and **(C)** IFN-y in the unstimulated and stimulated plasma of both healthy and LTBI participants. IP-10 was measured in ng/mL and INF-y was measured in pg/mL. *p* < 0.05 was considered statistically significant. Data was analyzed using one-way ANOVA and followed by a multiple comparisons test using Tukey’s test. The errors bars show the median and IQR.

**FIGURE 5:**
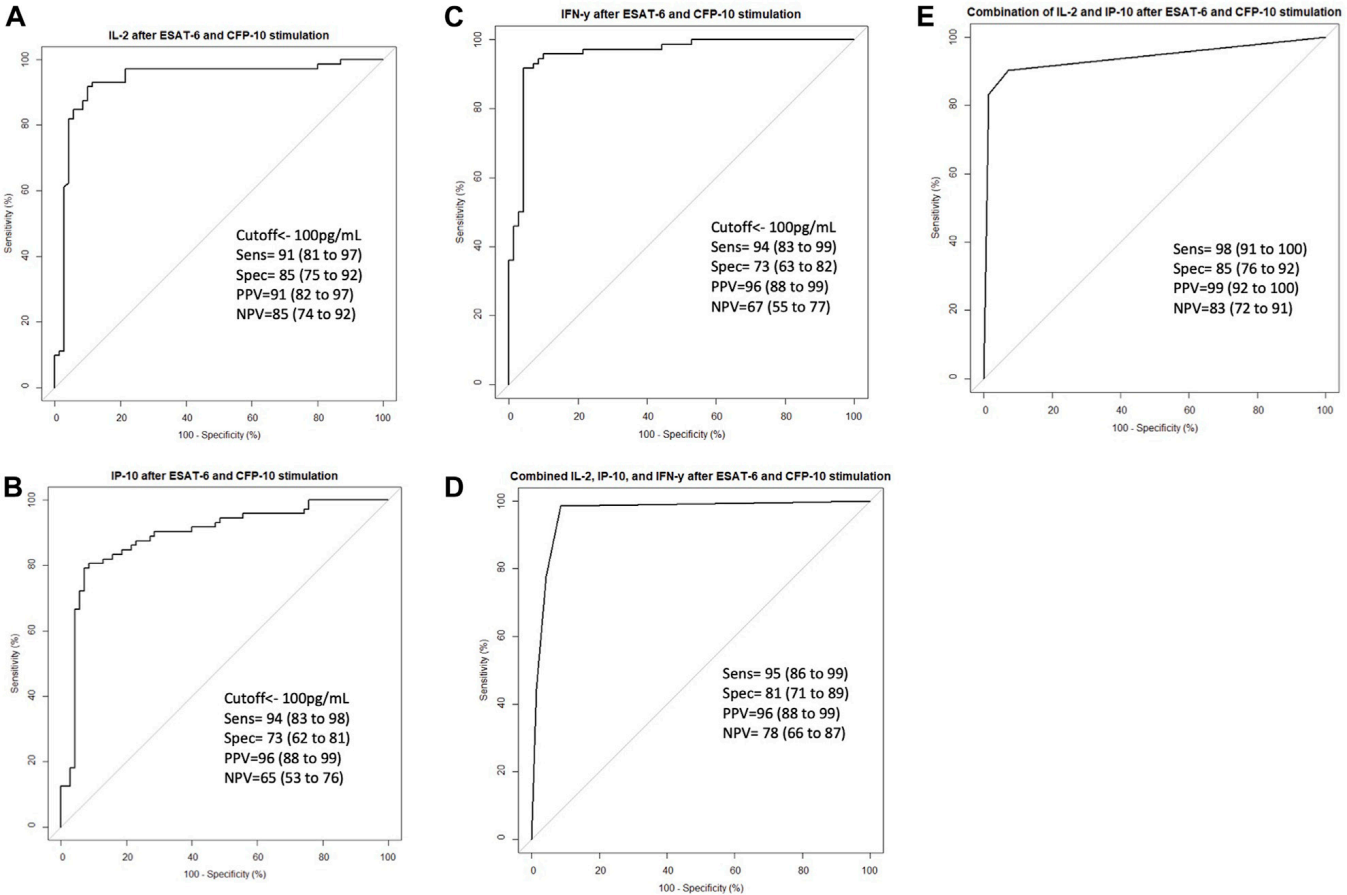
ROC curve characteristics for IL-2, IP-10, IFN-γ and combination of IL-2, IP-10 and INF-γ discriminatory markers of LTBI. ROC curves indicating AUC, sensitivity, specificity, PPV, and NPV of **(A)** IL-2, **(B)** IP-10, **(C)** INF-γ, **(D)** Combined IL-2, IP-10 and INF-γ, and **(E)** combined IL-2 and IP-10 in stimulated Healthy versus stimulated LTBI samples. Receiver operator characteristics (ROC) curve analysis of IL-2, IP-10 and INF-γ as biomarkers of LTBI were performed using pROC package. Summary receiver opeprator characteristics curves for combined IL-2, IP-10 and INF-γ and combined IL-2 and IP-10 were performed using pROC package in R software version 4.2.1 to define marker combinations of the tested stimulated plasma cytokines and chemokines.

To validate the effect of ESAT-6 and CFP-10 stimulation on the release of the cytokines that showed high discriminatory power, based on ROC characterisitics, between LTBI and healthy individuals, we measured and compared IL-2, IP-10 and IFN-γ levels in stimulated and unstimulated plasma samples from 70 LTBI and 72 healthy participants. There were elevated IL-2, IP-10 and IFN-γ levels in stimulated LTBI plasma compared to stimulated plasma of the healthy participants (*p* < 0.0001). Stimulated LTBI plasma IP-10, IL-2 and IFN-γ levels were also significantly higher than unstimulated plasma LTBI levels (*p*-value =<0.0001, <0.00001, and <0.0001), as shown in (Figures 4A–C). This indicates that the *M*.*tb* specific antigens (ESAT6 and CFP10) lead to cytokines production in cells that have memory of *M*.*tb*. There was no significant difference observed in unstimulated plasma IL-2, IP-10 and IFN-γ levels between the LTBI and healthy group (Figure 4). We further performed ROC analyses of IP-10, IL-2 and IFN-γ from ELISA data comparisng Healthy and LTBI samples (Figures 5A–C). For IL-2 and IFN-γ, we observed cut-off value of 100 pg/mL. IP-10 cut-off value was also 100 pg/mL. We noted the difference in the IP-10 cut-off value between this cut-off with the cut-off value of 300 pg/mL observed in the Bioplex assays (Figure 3C). This discrepancy could be due to the difference in the sample numbers, as the first cut-off of 300 pg/mL is based on bioplex data comparing 40 samples (20 healthy versus 20 LTBI), while IP-10 cut-off of 100 pg/mL in Figure 5B was based on comparison of higher sample size of 142 (72 healthy versus 70 LTBI) using ELISA assay. Analyses of diagnostic accuracy of combined IP-10, IL-2 and IFN-γ on a combined receiver operator characteristic revealed that the sensitivity of the combined three cytokines 95% (86–99) and combined specificity of 81% (71–89); (PPV = 96%; NPV = 78%) (Figure 5D), which were higher than individual IFN-γ sensitivity of 94% (83–99) and specificity of 73% (63–82) (PPV = 96%; NPV = 67%) respectively (Figure 5C). The combined IP-10 and IL-2 had sensitivity of 98% (91–100) and specificity of 85% (76–92), (PPV = 99%; NPV = 83%) (Figure 5E). These results indicate that IP-10, IL-2 and IFN-γ may have a potential as combined dagnostic biomarkers of LTBI.

## Discussion

At present, the diagnosis of LTBI is based on IGRA assays and TST; however, these tests may require improvement as they do not differentiate between individuals with active TB and LTBI, and IGRA performance may be affected by immunosuppression (Trajman et al., 2013; Shim, 2014). This study evaluated ESAT-6 and CFP-10 stimulated plasma samples for analyses of 27 cytokines and chemokines as potential biomarkers to distinguish between healthy and LTBI individuals. Previous studies have shown that stimulation of whole blood with TB antigen such as an ESAT-6 and CFP-10 may be used to induce *M.tb* specific immune response through the production of cytokines and chemokines (Suzukawa et al., 2016).

Suzukawa et al. (2016) identified IL1ra, IL2, IP-10 and MIP-1b as biomarkers that distinguish active TB from LTBI individuals in ESAT-6 and CFP-10 stimulated plasma. This study demonstrated that IL-1ra, IL-2, IP-10, and MIP-1b as well as IL-13 and IFN-γ were significantly increased in ESAT-6 and CFP-10 stimulated plasma levels of participants with LTBI compared to healthy individuals.

Furthermore, we and others have previously reported that cytokines and chemokines such as IL1ra, IL-6 and IP-10 in unstimulated plasma did not distinguish LTBI from healthy individuals, but LTBI from active TB (Schutz et al., 2019; Fisher et al., 2022). The current study differs from our previous approach, in that we stimulated whole blood with ESAT-6 and CFP-10 before detection of the cytokines.

Our study reported consistent results of significant increase in IL1ra and IL-2 in stimulated plasma of individuals with prior exposure to *M.tb*. IL-1ra is a cytokine that regulates pro-inflammatory responses of IL-1, through binding to their common receptor IL-1r1 (Schneider et al., 2021). Although mean IL-1ra of the LTBI population was higher than those of control participants, there were other control participants that had similarly high values. This variability in IL-1ra amounts could explain the lower ROC values for this marker. IL-2, a pro-inflammatory cytokine that is produced by Th-1 cells, effectively participates in the activation of T cells to produce tumor necrotic factor (TNF) and IFN-γ and enhances the cytolytic activity of natural killer cells (Abbas et al., 2018; Damoiseaux, 2020).

A previous study by Tientcheu et al. (2016) reported significantly higher level of IL-13 in *Mycobacterium Africanum* compared to *M.tb* in unstimulated plasma. In this study we observed significantly increased IL-13 levels after ESAT-6 and CFP-10 stimulation in LTBI participants. IL-13 is an anti-inflammatory cytokine, produced by Th2 cells and plays a role in the induction and maintenance of IgE production and allergic responses (de Vries, 1998). Future studies could look at the influence of various mycobacterial strains on cytokine production, which will aid in development of strain specific biomarkers.

In agreement with previous studies, we observed elevated IP-10 levels in LTBI individuals after stimulation with ESAT-6 and CFP-10 compared to healthy individuals (Borgström et al., 2012; Jeong et al., 2015; Comella-Del-Barrio et al., 2019). Biraro et al. (2016) previously showed that IP-10 performed well in differentiating contacts with either latent or active TB from those who were uninfected in Uganda. Our study corroborates these findings and the potential of IP-10 as a potential biomarker of LTBI (Biraro et al., 2016). IP-10 is secreted from cells stimulated with type I and II IFNs and LPS, and plays an important role in recruiting activated T cells into sites of tissue inflammation (Blauenfeldt et al., 2018). Several studies have shown the potential of IP-10 as biomarker for TB (Yassin et al., 2011). Given the variability and function of IFN stimulated innate immune cells that release IP-10, such as monocytes and macrophages, the performance of IP-10 alone could be heterogeneous and influenced by other underlying inflammatory conditions. Future studies could look into the influence of variable innate immune responses on the potential of IP-10 as an LTBI biomarker.

MIP-1b is produced by macrophages, active NK cells and T cells and play a role in activating human granulocytes (neutrophils, eosinophils, and basophils) which can lead to acute neutrophilic inflammation. This involves the production and release of pro-inflammatory cytokines like IL-1, IL-6 and TNF from fibroblasts and macrophages (Tam et al., 1996).

The combined ROC analyses show an improved sensitivity and specificity compared to IFN-y alone. Thus, our data shows that the combination of IL-2, IP-10 and IFN-y has a potential to improve IGRA alone for diagnosis of LTBI. The limitation of the study is that we could not test the performance of the combined cytokines in distinguishing LTBI from TB, or distinguishing Mtb from other non-tuberculous mycobacteria. Future studies could test the performance of the combined cytokines in such conditions.

Our data suggests that stimulating the whole blood with ESAT-6 and CFP-10 may be used to recall CD4 and CD8 T cell memory responses and the expression of certain biomarkers that may be used to distinguish LTBI from healthy individuals. Previous work has reported an IGRA based test that involves simultaneous detection of CD4 and CD8 T cell responses (Petruccioli et al., 2022). We hypothesize that IL-2, and IP-10, may serve as potential biomarkers for point of care to diagnosis of LTBI and could also be explored further as potential indicators of disease progression in those that are at risk of developing active disease. The high diagnostic accuracy observed in our ROC analysis for IL-2 and IP-10 indicate the potential of these cytokines as diagnostic tools that may be used together with IGRAs to increase the power of LTBI diagnosis. Interestingly, the combined IP-10 and IL-2 test appear to be more robust than the assay that combines IFN-γ, IP-10, and IL-2, suggesting that the combination of these two biomarkers, alone, could potentially serve as an alternative to the standard IGRA as an LTBI test. With future clinical validation studies, the combined test could provide an alternative to address some of the limitations associated with IFN-γ release assay, including in immunosuppressed individuals. The value of the current work lies in the setting in which the study is performed, particularly given the unique very high LTBI prevalence and high rates of HIV co-infection in KwaZulu Natal Region of South Africa (Wong et al., 2021; Mthembu et al., 2023). Future work could also evaluate the performance of the combined cytokines in detection of LTBI during HIV-TB co-infection.

## Data Availability

The original contributions presented in the study are included in the article/Supplementary Material, further inquiries can be directed to the corresponding author.
