# Supplementary material for: Combined analysis of host IFN-γ, IL-2 and IP-10 as potential LTBI biomarkers in ESAT-6/CFP-10 stimulated blood
Source: Front Mol Med. 2024 Jan 26;4:1345510. doi: 10.3389/fmmed.2024.1345510 (PMC11285608; doi:10.3389/fmmed.2024.1345510)
Supplement: Supplementary file 1 [file Table1.DOCX]

**Supplemental Table 1:** Absolute protein abundance of all ESAT-6 and CFP-10 stimulated plasma cytokines and chemokines assessed in this study

| Circulatory markers | **Latent TB** | | **Healthy** | | **p-Value** |
| --- | --- | --- | --- | --- | --- |
|  | **Median** | **IQR** | **Median** | **IQR** |  |
| IL-1b | 282.4 | (91.23 – 583.3) | 297.6 | (105.4 – 707.2) | 0.8831 |
| IL-1**r**a | 13963 | (9773 – 16329) | 8400 | (6869 – 12908) | 0.0056 |
| IL-2 | 322.1 | (175.3 – 774.0) | 42.57 | (27.02 – 70.08) | <0.0001 |
| IL-4 | 17.77 | (9.398 – 19.66) | 15.51 | (9.968 – 19.86) | 0.7330 |
| IL-5 | 330.6 | (212.0 – 509.4) | 324.2 | (227.3 – 429.3) | 0.5969 |
| IL-6 | 146 | (265.2 – 4208) | 1660 | (952.3 – 2862) | 0.7179 |
| IL-7 | 8.210 | (6.140 – 9.893) | 8.210 | (6.140 – 8.210) | 0.6659 |
| IL-8 | 13649 | (12841 – 21280) | 13631 | (11210 – 14709) | 0.2469 |
| IL-9 | 410.2 | (373.7 – 443.9) | 420.1 | (379.3 – 437.2) | 0.8620 |
| IL-10 | 7.000 | (4.505 – 11.48) | 8.275 | (6.355 – 11.28) | 0.1390 |
| IL-12 | 35.96 | (18.37 – 44.93) | 30.23 | (23.19 – 43.26) | 0.9519 |
| IL-13 | 25.78 | (9.395 – 42.97) | 0.4650 | (0.4650 – 0.4650) | <0.0001 |
| IL-15 | 505.2 | (334.7 – 583.5) | 473.6 | (347.1 – 584.6) | 0.7736 |
| IL-17 | 85.85 | (41.07 – 115.6) | 86.70 | (46.11 – 116.7) | 0.8149 |
| Eotaxin | 15.00 | (13.27 – 21.45) | 16.22 | (11.94 – 23.36) | 0.6832 |
| FGF basic | 191.4 | (117.4 – 222.8) | 188.5 | (11.94 – 23.36) | 0.9467 |
| G-CSF | 3166 | (2780 – 3707) | 3362 | (2888 – 3544) | 0.9946 |
| GM-CSF | 19.52 | (12.38 - 30.23) | 16.90 | (12.34 – 24.25) | 0.5335 |
| IFN-y | 170.9 | (101.2 – 325.2) | 56.41 | (48.73 – 81.11) | <0.0001 |
| IP-10 | 4943 | (1366 – 7147) | 565.4 | (159.1 – 1034) | <0.0001 |
| MCP-1 | 1282 | (1080 – 1447) | 1076 | (910.2 – 1287) | 0.0596 |
| MIP-1a | 304.4 | (304.4 – 373.6) | 304.4 | (304.4 – 370.3) | 0.9025 |
| PDGF-bb | 1356 | (987.1 – 1700) | 1767 | (1211 – 2207) | 0.0369 |
| MIP-1b | 2144 | (1667 – 2503) | 1237 | (879.4 – 1801) | 0.0010 |
| Rantes | 10673 | (8949 – 13034) | 10673 | (10673 – 15026) | 0.4932 |
| TNF-a | 647.8 | (319.9 – 1539) | 490.1 | (305.7 – 870.9) | 0.5648 |
| VEGF | 442.0 | (303.6 – 502.9) | 435.5 | (345.3 – 504.3) | 0.9095 |
